# Skin Temperature as a Marker of Physical Fitness Profile: The Impact of High-Speed Running in Professional Soccer Players

**DOI:** 10.3390/sports13120443

**Published:** 2025-12-09

**Authors:** Victor-Luis Escamilla-Galindo, Armiche Vega-Ramos, Jose Luis Felipe, Antonio Alonso-Callejo, Ismael Fernandez-Cuevas

**Affiliations:** 1 Department of Nutrition and Sports Sciences, Universidad de La Rioja, 26006 Logroño, Spain; 2 Department of Research, ThermoHuman, 28030 Madrid, Spain; ismael.fernandez@thermohuman.com; 3 Department of Performance, Orenburg FC, 460008 Orenburg, Russia; armi.vega.r@gmail.com; 4 IGOID Research Group, Department of Physical Activity and Sport Sciences, University of Castilla-La Mancha, 45071 Toledo, Spain; joseluis.felipe@uclm.es; 5 Performance Analysis Department, UD Las Palmas, 35019 Las Palmas de Gran Canaria, Spain; 6 Department of Sports Sciences, Faculty of Medicine, Health and Sports, Universidad Europea de Madrid, 28670 Madrid, Spain; antonio.alonso@universidadeuropea.es; 7 Sports Department, Faculty of Sciences for Physical Activity and Sport (INEF), Universidad Politécnica de Madrid, 28040 Madrid, Spain; 8 Department of Performance, Real Madrid CF, 28055 Madrid, Spain

**Keywords:** infrared thermography, performance, monitoring, internal load, recovery

## Abstract

High-speed running (HSR) has the greatest physiological impact on soccer players. It is closely linked to neuromuscular fatigue and muscle damage post-match, emphasizing the role that load monitoring plays in both performance and recovery. The aim of this study was to examine the relationship between match locomotor demands and the relative change in skin temperature (*%ΔT*) following official matches. A professional soccer team was analyzed during 14 regular-season matches. Infrared thermography (IRT) assessments were conducted before the match and up to 36 h after the match. The analyzed regions included posterior protocols of the lower limb. The kinematic variables of the match were obtained through a GPS device. Players were classified into high- and low-load groups based on the median values of HSR distance (372 m) and total distance (9675 m). Linear mixed-effects models showed that players in the high HSR group (≥372 m) demonstrated greater post-match decreases in *%ΔT*, particularly in the hamstring region (β = −1.79 ± 0.54 °C, 95% CI: −2.87 to −0.72, *p* = 0.001, R^2^ = 0.18), with a moderate-to-large effect size (ES = 0.67). Total distance also explained temperature change in the hamstrings (β = −1.46 ± 0.73 °C, *p* = 0.04). These findings suggest that post-match skin temperature reduction is sensitive to high-intensity running exposure, supporting IRT as a complementary internal load monitoring tool.

## 1. Introduction

High-intensity movement patterns in soccer have increased in magnitude during matches in recent years [1,2]. These high-intensity actions have proven to be the most relevant in identifying those players with greater success in competitions [3,4,5]. These actions occur mostly during the moments closest to scoring a goal [6].

Also, high-intensity actions have the greatest physiological impact on soccer players and are associated with a neuromuscular fatigue response and cause more muscle damage post-match [5,7,8]. Thereby, the physical demands with the highest injury rate in soccer are those that occur at high-speed running (HSR) (>21 km/h), with the muscles of the lower limb being the most affected [9].

This physiological impact has consequences for the recovery time of soccer players’ status after match [10]. Usually, players take between 48 and 72 h to return to their previous physiological normal values [11,12,13]. The growing density of competitive matches, especially during congested schedules, has further highlighted the importance of accurately monitoring recovery processes to optimize performance and reduce injury incidence [14,15].

To quantify external load, Global Positioning Systems (GPS) have become an essential tool in elite soccer to control physical demands during training and competition [16]. Proper interpretation of these data allows practitioners to individualize training microcycles in order to reduce injuries and maximize player performance [17]. However, GPS metrics alone do not provide direct information about internal physiological responses, emphasizing the need to integrate complementary technologies to better understand the performance and recovery status in soccer players [18].

Traditionally, the assessment of internal load and recovery in soccer players has been performed using invasive technologies (blood biomarker samples) [13] or requires the subject’s willingness and may be intrusive (questionnaires and isometric or jump tests) [19]. In this context, infrared thermography (IRT) has emerged as a promising non-invasive, contact-free, and safe alternative to monitor physiological status through skin temperature measurement as an objective alternative to measure internal load [20]. Under resting conditions, players typically maintain a stable thermal balance. However, the metabolic and mechanical stress of training and competition could potentially induce localized or systemic thermal changes that reflect underlying physiological processes [21].

Recent studies have explored the relationship between external workload (analyzed with GPS) and internal thermal responses (analyzed with IRT), with significant findings for changes in skin temperature asymmetries that may indicate localized fatigue, inflammation, or incomplete recovery [22,23]. Despite these advances, the use of IRT as a reliable marker of post-match recovery in elite soccer remains insufficiently validated. Specifically, few studies have simultaneously analyzed GPS-derived external load and microcycle-by-microcycle thermographic responses after competition, and the potential link between high-intensity actions and post-match responses is still unclear [24,25,26]. Furthermore, thermal responses have not been analyzed in parallel with objective high-intensity metrics, such as HSR meters, which limit the ability to interpret thermographic changes within the context of match demands.

Therefore, this study aimed to evaluate the association between high-intensity movement patterns, particularly HSR, and distance covered with variations in skin temperature responses to matches in professional soccer players during a period of the regular season. It was hypothesized that players who perform a higher volume of HSR and cover greater distances would show a different relative change in skin temperatures after the match than those who run less, reflecting a greater physiological load and delayed recovery.

## 2. Materials and Methods

### 2.1. Participants and Inclusion Criteria

The study design was longitudinal observational, aiming to analyze changes in skin temperature throughout the soccer season in a single professional team. A total of 24 players of a professional soccer team competing in the Russian Premier League (25.58 ± 4.42 years; 76.56 ± 6.7 kg; 181.23 ± 3.56 cm) were monitored using a GPS positioning system (Catapult Group International Ltd., Melbourne, Australia) during the regular 2022/2023 season. During the same period, players were evaluated in a baseline state before and after the matches using IRT. The inclusion criteria were as follows: having participated in at least one of the regular matches of the competition for a minimum of 60 min, being a field player, being available for pre- and post-match IRT measurements in the match they participated in, and not having suffered any injuries during the evaluation period.

### 2.2. Procedures

#### 2.2.1. Description of IRT Assessment

**Figure 1:**
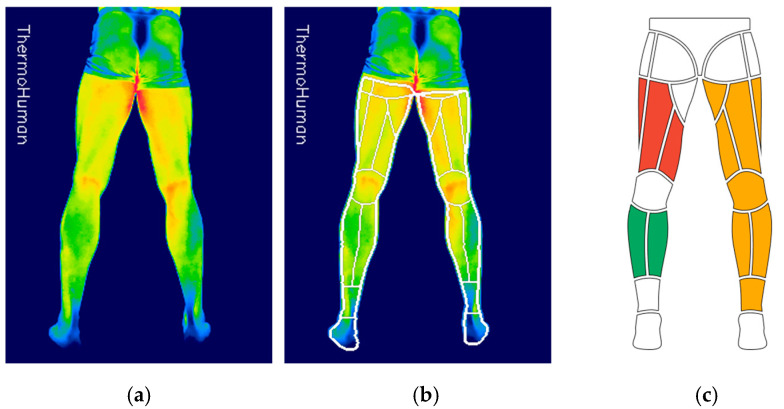
Skin temperature analysis protocol using ThermoHuman software. (**a**) Thermogram of the posterior leg. (**b**) Segmentation of regions of interest through software. (**c**) Grouped regions for analysis: red for hamstrings, green for calves, yellow for the postero-inferior (PI) region (right and left sides included in every grouped region).

A thermographic analysis was performed between 24 and 36 h before the match day (MD-1/MD-2) and between 24 and 36 h after the match day (MD + 1/2), with a baseline before training without any treatment or exercise performed by the player. An IRT-certified specialist technician performed image capture, meeting the requirements of the TISEM checklist [27], and players were instructed to avoid consuming stimulants, such as coffee or alcohol, maintain an 8 min acclimatization period, sit in an upright position perpendicular to the camera at a distance of 3 m with the camera at knee height. Shots were taken with an emissivity of 0.98 during the early morning hours when the players accessed the club facilities, where the room was climate-controlled with a temperature range between the assessments of (20 °C ± 2 °C) and a relative humidity of (53% ± 9%). They underwent thermal evaluation in sequential order, and their weight was also recorded. For image capture, a FLIR E-54 EST camera (Teledyne FLIR, Wilsonville, OR, USA) was used, and for image analysis, the ThermoHuman software, version 2.10 (Pema Thermo Group SL., Madrid, Spain) was employed. The software automatically extracted regions of interest by means of artificial vision algorithms that were previously validated [28], reporting on the average, minimum, and maximum temperature, and the asymmetry between contralateral regions from the lower limbs using two types of protocols: the anterior-inferior protocol and the postero-inferior (PI) protocol (Figure 1).

#### 2.2.2. Extraction of Thermal Regions Using the Software

For the purpose of this study, the means of three grouped regions were selected: all the regions on the back of the body, which included regions from the hamstrings to the Achilles tendon, were grouped and referred to as the PI region; the three regions located in the hamstring area were also grouped to perform a single analysis of that region (hamstring); and finally, the two calves regions were grouped to analyze a single combined region (calves). All these areas comprised the IRT variables (PI, hamstrings, and calves).

#### 2.2.3. Acquisition of GPS Data During the Season

For GPS analysis during matches, the players wore an individual GPS device during the match that recorded the kinetic and kinematic parameters developed. A total of 24 different players participated in the matches, completing at least 60 min of the match, which amounted to a total of (m = 115 samples). On average, each player contributed to 2.95 matches during the study period (with one player who only played one game and the player who played the most games playing 10 games). Missing data were retained as ‘NaN’ values and were not imputed; statistical analyses were performed only on complete cases for each variable. They recorded data such as distance covered (total distance (DIST); m), and distance covered (m) at speeds above 21 km/h (high-speed running; HSR). This technology has previously demonstrated its validity in measuring these kinematic variables [29].

To analyze the thermal response provoked by load, two groups were made for each load variable (total distance and HSR) based on the median value of each distribution. Each player who participated in over 60 min of a match was included for observation. Observations which covered a total distance lower than 9675 m (median value for total distance) were classified as −DIST (*n* = 54) and those who covered more were grouped as +DIST (*n* = 54). The same process was used to divide the sample in the HSR variable with a split value of 372 m (+HSR, *n* = 54; −HSR, *n* = 54). All groups have the same number of observations since, by median, group distribution is equal.

### 2.3. Statistical Analysis

For each of the IRT variables (PI, hamstrings, and calves), the relative temperature change (*ΔT*) between pre- and post-match was calculated, with *%ΔT* defined as (((post-match temperature − pre-match temperature)/pre-match temperature) × 100). Subsequently, normality testing using the Kolmogorov–Smirnov test was performed on the newly derived variables, confirming a normal distribution. Descriptive statistics (mean ± standard deviation) by group (HSR split and distance split) were calculated for the variables: minutes played, distance covered, HSR, and skin temperatures recorded in the three IRT variables.

To test associations while accounting for repeated measures within players, linear mixed-effects models (LMMs) were fitted separately for each IRT variable. Two primary LMMs were specified:

A linear mixed-effects model (MLM) was fitted with *%ΔT* as the outcome and minutes as a covariate and HSRgroup (+HSR vs. −HSR) as the main predictor. An intercept random effect for player was included to account for repeated measures:

*ΔT_ij_* = *β*_0_ + *β*_1_(*Minutes_ij_*) + *β*_2_(*HSRgroup_i_*) + *u*_0*j*_ + *ε_ij_*.

On the other hand, an analogous MLM with Distgroup (+DIST vs. −DIST) as main predictor, controlling for minutes and including player as a random intercept:

*ΔT_ij_* = *β*_0_ + *β*_1_(*Minutes_ij_*) + *β*_2_(*Distgroup_i_*) + *u*_0*j*_ + *ε_ij_*.

Model parameters reported are unstandardized *β* coefficients and two-sided *p*-values; statistical significance was set at α = 0.05, where *u*_0*j*_ is the random intercept for the player j and *ε_ij_* is the residual error. Minutes were included as a covariate in both models.

In addition, Cohen’s d effect sizes were computed to quantify the magnitude of the group differences. For the HSR and distance splits, Cohen’s d was calculated as the difference in group means of *%ΔT* divided by the pooled standard deviation. Effect sizes were classified as small (≈0.2), medium (≈0.5), and large (≈0.8), following conventional thresholds. There was a 95% confident interval of the differences (95%CI). Conditional R2 and marginal R2 were calculated to inform the explained variance of the model, taking both the fixed and random effects into account. All statistical analyses were performed in Python, version 3.10 (pandas, numpy, statsmodels, Pingouin).

## 3. Results

Table 1 shows descriptive values (mean ± sd) for both groups of each load variable and the *%ΔT* as a percentage between pre-match and post-match.

Linear mixed-effects models were applied to examine the influence of HSR exposure on the *%ΔT* across the postero-inferior region (PI), hamstrings, and calves, with minutes played included as a covariate and player as a random intercept (Table 2).

The results indicated that players classified in the +HSR group (≥372 m) showed greater decreases in skin temperature compared to players in the −HSR group, who showed an increase in the *%ΔT*. It is observed that for each player throughout the season, there was a significant negative response every time more HSR actions were performed. For the hamstring region, the +HSR group also exhibited the greatest relative decrease in temperature (β = −1.79 ± 0.54 °C, 95% CI: −2.87 to −0.72, *p* = 0.001), with a conditional R^2^ of 0.18. In this case, the effect size is moderate to large (ES = 0.67), showing the hamstring region has the most pronounced *%ΔT* after the matches. Across all regions, the coefficient for minutes played was negative but small and non-significant, indicating that match duration contributed minimally to temperature change compared to HSR load.

A second set of mixed-effects models evaluated whether total distance covered influenced relative skin temperature change, again controlling for minutes played. The high-distance group (≥9675 m) exhibited only a significant post-match relative temperature decrease in the hamstring region, suggesting that only this region could be affected by a high load (Table 3).

In the hamstring region, higher total distance was associated with a significant decrease in temperature (β = −1.46 ± 0.73 °C, 95% CI: −2.90 to −0.30, *p* = 0.04) with a conditional R^2^ of 0.09. In this case, the effect size is small (ES = 0.36), indicating that the total distance variable has less impact than the meters to HSR.

Again, minutes played did not significantly predict temperature change, suggesting that movement intensity (HSR and distance load) was more relevant than total match exposure time.

**Figure 2:**
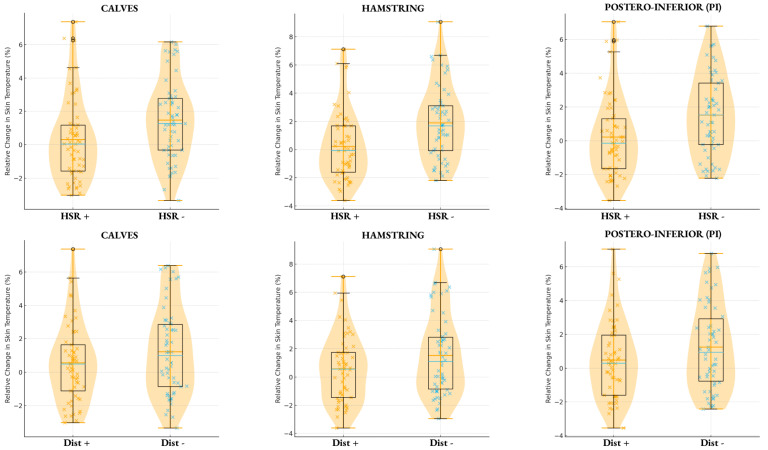
Violin and boxplot composite of *%ΔT* for the postero-inferior region (PI), hamstrings, and calves, comparing players in the high-load and low-load groups for HSR (>372 m (HSR+) or <372 m (HSR-)) and total distance covered (>9675 m (DIST+) or <9675 m (DIST-).

Figure 2 displays combined violin and boxplot distributions for pre-to-post *%ΔT* across the three analyzed regions (PI, hamstrings, and calves), comparing high vs. low HSR groups and high vs. low total distance groups. In all regions, the +HSR group showed lower median and mean temperature values following match play, consistent with the mixed-model results. Meanwhile, only +DIST in hamstrings showed lower median and mean temperature values following match play.

Overall, the findings indicate that match-related locomotor demands, particularly HSR exposure, have a meaningful impact on post-match *%ΔT*. Players performing greater HSR distances consistently demonstrated larger relative decreases in temperature, most notably in the hamstring region, where the effect was moderate to large. In contrast, total distance covered showed a weaker influence, with significant effects only detected in the hamstrings and with smaller effect sizes.

## 4. Discussion

The aim of this study was to examine the effects of match-related locomotor demands (HSR and total distance) on post-match changes in skin temperature during a professional soccer season, using *%ΔT* as an intra-player indicator.

One of the most determining variables, both in individual and team success, and with significant physiological impact on the player, is HSR. This action has been used in the literature to differentiate players with more success during the competition [4]. Furthermore, it is a risk factor for injury as it exposes players to high neuromuscular demands, particularly in the hamstring region [7,9]. Therefore, the control and monitoring of HSR have gained great importance in the planning of competitive microcycles [3].

In this sense, to the knowledge of the authors, this is the first study with professional soccer players to understand how competition match-related locomotor demands affect the skin temperature of the players during a regular season.

Previous research analyzing the response of athletes to long-duration aerobic stimuli has been evaluated using IRT, and the results have shown an increase in skin temperature in the days following competition [24]. Other studies have assessed multiple exposures to strength, speed, or endurance training, and all of them have shown an increase in skin temperature within the next 8 h [20]. Furthermore, competitions in hot environments have been found to lead to an increase in skin temperature in the subsequent days [30]. This means that mean temperature usually increases after competition. However, these studies do not distinguish between intra-player variations and do not analyze whether different loads elicit similar variations.

Previous studies analyzed a professional soccer team in a baseline state and after a 16-week competitive period, allowing for 72 h of recovery at the end of the competitive period. The results they obtained show an increase in skin temperature between the two periods, with competitive load being a factor in the temperature increase [25]. These findings are consistent with the significant data from the group −HSR and the −DIST group, in which the *%ΔT* is higher than +HSR and +DIST groups.

The perspective of this research is to consider high-intensity HSR load during the match as a mediator of skin response. The results include two measurements taken at very close time points, from 36 h before to 36 h after the match, aiming to minimize external factors that may affect changes in ambient temperature. Analyzing absolute temperatures may have been a limiting factor in other studies.

The main finding was that exposure to high-speed running (HSR ≥ 372 m) consistently predicted a greater post-match decrease in skin temperature, particularly in the hamstring region. This effect remained after controlling for minutes played, which showed a negligible association with *%ΔT* across all regions. In contrast, total distance ≥ 9675 m showed a smaller and region-specific effect, with only the hamstring region showing a statistically significant decrease in *%ΔT*. The effect size for total distance was small, indicating lower practical relevance when compared to HSR. In summary, these results suggest that HSR is a more significant indicator of acute physiological cost than total match distance, highlighting *%ΔT* hamstring as a key variable in monitoring internal load for performance monitoring tools in professional soccer.

The hamstring region appears to be the most responsive to this type of stimulus during the match, especially considering the HSR variable. These results regarding the temperature variation in the hamstring region after the match are novel, although there is previous research on sprints and hamstring temperature indicating that this region’s thermoregulation is affected when external fatigue is imposed through high-speed running work [23]. Therefore, when energy substrates are fully depleted, athletes’ skin temperature decreases [31]. Additionally, different types of fatigue can affect the nervous, muscular, and/or metabolic systems to varying degrees of *%ΔT* [32]. The type of high-intensity stimulus that will generate fatigue could condition the different responses in the relative change in temperature after matches.

As practical applications, the direction (positive or negative) and the magnitude of the observed effects suggest that relative skin temperature changes, especially in the hamstring region, could provide a non-invasive indicator. This indicator could help to contextualize how players respond to high-intensity loads during matches within athlete monitoring systems. It is important to note that the interpretation of *%ΔT* must be individualized, given the random intercept structure that indicates inter-player variability. Therefore, thermal responses should be assessed longitudinally and for each player, rather than comparing them between players or generalizing them across teams.

The limitations of this study include the inability to control the soccer team throughout the entire season due to the idiosyncrasies of the competitive calendar, which includes a break during the winter period. Additionally, due to the anonymous nature of the sample, neither the position nor the tactical model have been taken into account in the analyses, which could be a confusing factor for obtaining meters at high intensity and which could be considered as covariate, potential bias, or limitations in generalizing the findings. The results should be interpreted within the context of a single professional team and a non-consecutive sample.

Despite these limitations, we consider that the study outcomes may be of great utility for technical soccer staff to have an objective criterion through thermal behavior of the body to make decisions in order to individualize the load during the microcycle. Future work should examine whether similar patterns are observed across different competitive leagues, tactical contexts, position of the player, or a complete season.

## 5. Conclusions

This study evaluated the relative skin temperature change following official matches in professional soccer players and examined its relationship with match locomotor demands. From a monitoring perspective, the assessment of *%ΔT* through IRT after competition may serve as an internal load indicator. Among the analyzed regions, the hamstring region appears to be the most responsive to high-intensity demands, decreasing the temperature after the match, suggesting its relevance for individualized follow-up during congested schedules or periods of high external load.

Overall, incorporating IRT into post-match assessments may complement other monitoring tools, helping sport scientists and performance staff contextualize high-intensity movement patterns and guide individualized recovery and training decisions throughout the competitive season.

## Figures and Tables

**Table 1:** Descriptive values (mean ± sd) for total distance and HSR with the *%ΔT* as percentage differences between pre-match and post-match for the regions of the postero-inferior protocol, hamstring, and calves.

		Load Variable (DIST in km; HSR in m)	Minutes Played (min)	Pre PI ST (°C)	Post PI ST (°C)	PI (*%ΔT*)	Pre Hamstrings ST (°C)	Post Hamstrings ST (°C)	Hamstrings (*%ΔT*)	Pre Calves ST (°C)	Post Calves ST (°C)	Calves (*%ΔT*)
DIST	+DIST	10.5 ± 0.6	97.5 ± 3.5	31.82 ± 0.6	31.97 ± 0.6	0.4 ± 2	31.9 ± 0.7	32.1 ± 0.9	0.5 ± 2	31.9 ± 0.7	32 ± 0.7	0.5 ± 2
	−DIST	8.3 ± 1.1	82.60 ± 13.57	31.5 ± 0.6	31.96 ± 0.7	1.2 ± 2	31.6 ± 0.7	32.1 ± 0.8	1.5 ± 3	31.6 ± 0.6	31.9 ± 0.7	1.2 ± 3
HSR	+HSR	584.79 ± 176.32	91.76 ± 11.16	31.70 ± 0.6	31.76 ± 0.7	0.1 ± 2	31.80 ± 0.6	31.79 ± 0.8	−0.1 ± 3	31.65 ± 0.74	31.74 ± 0.71	0.3 ± 2
	−HSR	269.70 ± 66.48	88.23 ± 13.44	31.69 ± 0.5	32.17 ± 0.6	1.5 ± 2	31.74 ± 0.7	32.3 ± 0.7	1.8 ± 3	31.75 ± 0.57	32.21 ± 0.61	1.5 ± 2

PI *%ΔT*: Relative temperature change extracted from IRT assessment for the postero-inferior protocol.

**Table 2:** Results of the Linear Mixed Effects Model on *%ΔT* for the regions of interest split by HSR analyzed with thermography, and with minutes played as a covariate.

HSR (m)							
	Intercept	*β*_1_ Minutes	*β*_2_ HSR Group	CI 95%	*p*-Value	Effect Size (Cohen’s d)	R^2^ (Marginal = Conditional)
PI (°C) *ΔT*	−2.57 × 10^−5^	−0.006	−1.46 ± 0.52	[−2.49, −0.43]	0.005 *	0.56	0.13
Hamstring (°C) *ΔT*	−2.69 × 10^−5^	−0.009	−1.79 ± 0.54	[−2.87, −0.72]	0.001 *	0.67	0.18
Calves (°C) *ΔT*	−2.74 × 10^−5^	−0.007	−1.23 ± 0.55	[−2.32, −0.13]	0.027 *	0.48	0.09

* *p* < 0.05. Cond. R^2^: Conditional R^2^.

**Table 3:** Results of the Linear Mixed Effects Model on *%ΔT* for the regions of interest split by total distance analyzed with thermography, and with minutes played as a covariate.

Total Distance (m)							
	Intercept	*β*_1_ Minutes	*β*_2_ Dist Group	CI 95%	*p*-Value	Effect Size (Cohen’s d)	R^2^ (Marginal = Conditional)
PI (°C) *ΔT*	−2.62 × 10^−5^	0.01	−1.26 ± 0.69	[−2.62, 0.09]	0.06	0.32	0.07
Hamstring (°C) *ΔT*	−2.77 × 10^−5^	0.01	−1.46 ± 0.73	[−2.90, −0.3]	0.04 *	0.36	0.09
Calves (°C) *ΔT*	−2.79 × 10^−5^	0.003	−0.82 ± 0.73	[−2.26, 0.62]	0.26	0.26	0.03

* *p* < 0.05. Cond. R^2^: Conditional R^2^.

## Data Availability

The current study shares its research data in the Mendeley repository: “Skin Temperature Football Players during a season”, Mendeley Data, V1, https://doi.org/10.17632/spkmxfsmhv.1.

## Abbreviations

The following abbreviations are used in this manuscript:

HSR	High-speed running
GPS	Global Positioning Systems
IRT	Infrared thermography
PI	Postero-inferior
*ΔT*	Relative temperature change
MD	Match day
DIST	Total distance
